# Association of Trabecular Bone Score with Inflammation and Adiposity in Patients with Psoriasis: Effect of Adalimumab Therapy

**DOI:** 10.1155/2016/5747852

**Published:** 2016-05-12

**Authors:** José L. Hernández, Raquel López-Mejías, Ricardo Blanco, Trinitario Pina, Sheila Ruiz, Isabel Sierra, Begoña Ubilla, Verónica Mijares, Marcos A. González-López, Susana Armesto, Alfonso Corrales, Enar Pons, Patricia Fuentevilla, Carmen González-Vela, Miguel Á. González-Gay

**Affiliations:** ^1^Bone Metabolism Unit, Department of Internal Medicine, Hospital Marqués de Valdecilla-IDIVAL, University of Cantabria, RETICEF, Avenida de Valdecilla, s/n, Santander, 39008 Cantabria, Spain; ^2^Division of Rheumatology, Hospital Marqués de Valdecilla-IDIVAL, University of Cantabria, Avenida de Valdecilla, s/n, Santander, 39008 Cantabria, Spain; ^3^Division of Dermatology, Hospital Marqués de Valdecilla, Avenida de Valdecilla, s/n, Santander, 39008 Cantabria, Spain; ^4^Department of Pathology, Hospital Marqués de Valdecilla, Avenida de Valdecilla, s/n, Santander, 39008 Cantabria, Spain

## Abstract

Studies on trabecular bone score (TBS) in psoriasis are lacking. We aim to assess the association between TBS and inflammation, metabolic syndrome features, and serum adipokines in 29 nondiabetic patients with psoriasis without arthritis, before and after 6-month adalimumab therapy. For that purpose, adjusted partial correlations and stepwise multivariable linear regression analysis were performed. No correlation was found between TBS and disease severity. TBS was negatively associated with weight, BMI, waist perimeter, fat percentage, and systolic and diastolic blood pressure before and after adalimumab. After 6 months of therapy, a negative correlation between TBS and insulin resistance (*p* = 0.02) and leptin (*p* = 0.01) and a positive correlation with adiponectin were found (*p* = 0.01). The best set of predictors for TBS values at baseline were female sex (*p* = 0.015), age (*p* = 0.05), and BMI (*p* = 0.001). The best set of predictors for TBS following 6 months of biologic therapy were age (*p* = 0.001), BMI (*p* < 0.0001), and serum adiponectin levels (*p* = 0.027). In conclusion, in nondiabetic patients with moderate-to-severe psoriasis, TBS correlates with metabolic syndrome features and inflammation. This association is still present after 6 months of adalimumab therapy. Moreover, serum adiponectin levels seem to be an independent variable related to TBS values, after adalimumab therapy.

## 1. Introduction

The trabecular bone score (TBS) is a new developed method used to indirectly evaluate bone microarchitecture, therefore providing skeletal data not captured from the standard dual-energy-X-ray absorptiometry (DXA) [1]. It consists of a texture parameter that evaluates pixel-gray level variations in the projected lumbar spine DXA image. Thus, TBS may be considered as an overall descriptor of bone quality, and lower values have been associated with worse bone structure and high risk of fractures [2]. Moreover, the utility of TBS in the fracture risk assessment or treatment onset, in patients with osteoporosis, has been addressed in a recent position study by The International Society for Clinical Densitometry [3].

In a cross-sectional, population-based study from Korea, lumbar TBS values were lower in patients with type 2 diabetes mellitus. Moreover, participants with low TBS values had more commonly insulin resistance and raised serum high-sensitivity C-reactive protein (hs-CRP) levels than those with high values, irrespective of age and body mass index (BMI) [4].

Psoriasis is associated with increased cardiovascular risk [5]. Patients with psoriasis have frequently metabolic syndrome features, such as insulin resistance, dyslipidemia, hypertension, or obesity [6]. Furthermore, inflammation plays a crucial role in the development of atherosclerosis in these patients [7]. Adipokines have also been reported to influence the increased cardiovascular risk in systemic disorders. In this sense, we have previously published that, in nondiabetic patients with moderate-to-severe psoriasis, leptin correlates with some metabolic syndrome features, whilst resistin correlates with inflammation and disease severity. Besides, we have also found that antitumor necrosis factor-*α* (anti-TNF-*α*) agents may improve insulin resistance in patients with moderate-to-severe psoriasis [8], although no significant changes in serum leptin or resistin concentrations after 6 months of adalimumab therapy were found [6].

Taking into account these considerations, we aimed to explore the possible association between TBS and inflammation or metabolic syndrome features in nondiabetic patients with moderate-to-severe psoriasis without arthritis, before and after adalimumab therapy. We also analyzed the effect of serum adipokines on TBS values in these patients.

## 2. Materials and Methods

### 2.1. Patients and Treatment Scheme

Patients with moderate-to-severe psoriasis and without a clinical pattern of psoriatic arthritis were consecutively enrolled, over an 18-month period from the Dermatology Outpatient Clinic of the University Hospital Marqués de Valdecilla, a tertiary-care center in Northern Spain. Recruitment protocol has been previously described [6–8]. Patients with diabetes, kidney disease, hypertension (systolic blood pressure (SBP) >140 mm Hg or diastolic blood pressure (DBP) >90 mm Hg or taking antihypertensive drugs), or BMI ≥ 35 Kg/m^2^ were excluded from the study. None of the patients had any bone disease or were on drugs affecting bone or lipid metabolism.

All patients received adalimumab (Humira, Abbot Laboratories SA, Madrid, Spain), 80 mg subcutaneously at week 0 followed by 40 mg every other week, starting 1 week after the initial dose. The study protocol was approved by the local institutional ethics committee of Cantabria (Spain), and it was in accordance with the ethical standards outlined in the Declaration of Helsinki. Written informed consent was obtained for all participants.

### 2.2. Clinical Assessment

Main demographic and clinical features were obtained at baseline (before adalimumab onset) and after six months of therapy, according to a predefined protocol. Height and weight were measured with participants wearing light indoor clothing but without shoes. BMI was defined as weight (kg) divided by squared height (m^2^). Waist circumference was measured in centimeters at a level midway between the lower rib margin and iliac crest after breathing out, with a flexible tape all around the body, in an erect position with feet together. Smoking habits were assessed by asking whether the participant was currently smoking, had never smoked, or was an ex-smoker. Regarding alcohol consumption, subjects with an intake of ≥20 g/day were considered as current drinkers. Blood pressure was measured in the nondominant arm using a calibrated oscillometric sphygmomanometer Omron 705IT (HEM-759-E, Omron Corporation, Kyoto, Japan), with a cuff of the appropriate size following standard recommended procedures [9]. Two readings each for the SBP and DBP were taken in a 5 min interval after 5 minutes' rest. The average of the two readings was used for the data analysis. If the two measurements differed by more than 5 mm Hg, then an additional reading was taken, and the average of the three readings was used. In each subject, body composition was measured by DXA (Hologic QDR 4500, Bedford, MA, USA). Quality control was performed according to the usual standards [10]. TBS measurements were retrospectively made on lumbar DXA files, using TBS iNsight Software (Medimaps) by the same expert technician, blind to all clinical parameters.

Disease activity was assessed by means of the percent of body surface area (BSA) affected, Psoriasis Area and Severity Index (PASI), Psoriatic Arthritis Screening and Evaluation questionnaire (PASE), Nail Psoriasis Severity Index (NAPSI), and physician's global assessment (PGA) of disease severity. Moderate-to-severe psoriasis was defined whether BSA was ≥10% and/or PASI ≥ 10 [6, 8].

### 2.3. Laboratory Measurements

Blood samples were taken after a 12-hour overnight fast between 08:00 am and 10:00 am for two separate visits: at baseline and 6 months after the onset of adalimumab. Routine parameters were measured by standard automated methods on an ADVIA 2400 Chemistry System (Siemens, Los Angeles, USA). Low density lipoprotein cholesterol (LDL-C) was calculated by the Friedewald formula. Apolipoprotein-A (apo-A), Apo-B, lipoprotein a (Lpa), homocysteine, and high-sensitive C-reactive protein (hs-CRP) were determined by immunonephelometry (Behring Nephelometer Analyzer II, Behring Diagnostics, Marburg, Germany). Insulin was quantified by specific automated immunoassay (Liaison, DiaSorin, Stillwater, Minnesota, USA). Erythrocyte sedimentation rate (ESR) was determined using the Westergren method. The Homeostasis Model Assessment (HOMA) and the Quantitative Insulin Sensitivity Check Index (QUICKI) were used as noninvasive surrogate markers of insulin resistance and insulin sensitivity, respectively. Leptin serum levels were determined by a commercially available ELISA (Linco Research, St. Charles, MO, USA; Human Leptin ELISA Kit, EZHL-80SK; assay sensitivity = 0.135 ng/mL ±2 SD; intra- and interassay coefficients of variation: 3.7% and 4%, resp.) according to the manufacturer's instructions. Serum resistin levels were measured by ELISA (Linco Research, St. Charles, MO, USA; the assay sensitivity was 0.16 ng/mL, and the intra- and interassay coefficients of variation were <5% and <7%, resp.). Serum visfatin levels were measured by ELISA (minimum detectable concentration: 1.85 ng/mL; range: 0.1–1000 ng/mL; linear range: 1.85–19.5 ng/mL; intra- and interassay coefficients: 5%–12%) (Phoenix Pharmaceuticals, Inc., Burlingame, California, USA). Plasma adiponectin levels were measured by ELISA (EMD Millipore, St. Charles, Missouri, USA). The assay sensitivity was 0.2 ng/mL, and the intra- and intercoefficients of variation were 3.3% and 5.5%, respectively.

### 2.4. Statistical Analysis

Results were reported as mean ± standard deviation (SD) or as median and interquartile range (IQR), as appropriate. Mann-Whitney *U* test was performed to compare quantitative variables and Chi-square test or Fisher exact test to compare qualitative variables. Wilcoxon signed rank test was used for paired comparisons between baseline and 6-month values. Pearson partial correlation coefficient (*r*) was used to analyze the association of TBS with the studied variables, prior to adalimumab onset (at baseline) and after six months of treatment. Results were adjusted for age at the study onset, sex, and duration of the disease. Besides, a stepwise multivariable linear regression analysis was performed to ascertain the best set of predictors for TBS values, at baseline and after 6 months of therapy with adalimumab. Collinear diagnostics within variables were applied before the regression modeling. STATA 12/SE software (StataCorp, College Station, TX) was used in all the calculations. Differences were considered statistically significant at *p* < 0.05.

## 3. Results

We included 29 patients who completed a 6-month period of treatment with adalimumab. The main demographic and clinical features of these patients are summarized in Table 1. As previously reported [8], a significant reduction (*p* < 0.05 for each comparison) in all of the markers of disease activity and severity was observed at 6 months after the onset of adalimumab.

### 3.1. Association between TBS and Clinical Parameters, Adiposity, and Inflammation

At baseline, mean TBS values were 1.409 ± 0.097 in men and 1.484 ± 0.095 in women (*p* = 0.04). After 6 months of adalimumab, these values were 1.411 ± 0.123 and 1.487 ± 0.103, respectively (*p* = 0.07). TBS values were not statistically different between baseline and at 6 months of the anti-TNF-*α* drug treatment, neither in the overall group nor in men or women separately (Table 2). No differences were seen regarding TBS values and type of psoriasis.

No correlation was found between adjusted TBS values and the main indexes of disease severity, neither at baseline nor at 6 months (Table 3).

Although patients with BMI ≥ 35 kg/m^2^ were excluded, in our series of patients with moderate-to-severe psoriasis, TBS was negatively associated with weight, BMI, and waist perimeter at study onset. The administration of adalimumab did not change this relationship. In keeping with that, we found a statistically significant negative correlation between TBS and fat percentage measured by DXA at baseline and at 6 months of anti-TNF-*α* drug onset (Table 3).

Among lipid parameters, we only disclosed an inverse correlation between TBS and Castelli index (Table 3), although such an association was no longer significant after 6 months of treatment with adalimumab.

A statistically significant negative correlation between TBS and hs-CRP was also observed at baseline and after 6 months of therapy, once adjusted for sex, age at study onset, and disease duration (Table 3).

### 3.2. Association between TBS and Metabolic Syndrome Features Other Than Adiposity

We found a negative correlation between SBP and DBP and TBS values (Table 3). This association was present at baseline and also after 6 months of adalimumab therapy. Noteworthily, following 6 months of adalimumab therapy, a statistically negative correlation between TBS values and insulin resistance (measured by HOMA; *r* = −0.445; *p* = 0.02) and a positive one between TBS and insulin sensitivity (QUICKI; *r* = 0.497; *p* = 0.01) were found. These significant correlations were not seen prior to the onset of adalimumab (Table 3).

### 3.3. Association between TBS and Serum Adipokines

Adipokine serum levels did not correlate with TBS at baseline (Table 3). Nevertheless, after adjustment for sex, age at the onset of the study, and duration of psoriasis, a statistically significant negative correlation between adiponectin levels and TBS was observed following 6 months of biologic therapy (*r* = −0.489; *p* = 0.01). In this regard, plasma adiponectin concentrations showed a significant reduction following anti-TNF-*α* therapy (16630.1 ± 12646.9 ng/mL at baseline versus 9659.5 ± 4980.2 ng/mL after 6 months of adalimumab; *p* = 0.013).

Besides, after 6 months of biologic therapy, a negative correlation between levels of leptin and TBS was observed (*r* = −0.479; *p* = 0.01). In contrast, serum levels of resistin and visfatin did not show any significant difference following adalimumab therapy (Table 3).

### 3.4. Multivariable Predictors for TBS Values

In stepwise multiple regression analysis, the best set of predictors for TBS values in this series of nondiabetic patients with moderate-to-severe psoriasis were female sex (*β* = 0.372; *p* = 0.015), age at the study onset (*β* = 0.276; *p* = 0.05), and baseline BMI (*β* = −0.557; *p* = 0.001). The adjusted *R* ^2^ of the model was 0.54. The best set of predictors for TBS following 6 months of therapy with adalimumab were age (*β* = 0.432; *p* = 0.001), BMI (*β* = −0.600; *p* < 0.0001), and serum adiponectin levels (*β* = 0.289; *p* = 0.027). The adjusted *R* ^2^ of the regression model was 0.67.

## 4. Discussion

Bone quality assessment in patients with psoriasis with and without arthritis has been recently reported by Kocijan et al. [11]. They have assessed quantitative and microstructural bone changes in 30 patients with psoriasis and 50 with psoriasis arthritis (PsA) by means of high-resolution peripheral quantitative computed tomography (HR-pQCT) scans at the ultradistal and periarticular radius. They found that bone microarchitecture measurements (mainly trabecular volume and number of trabeculae) were lower in PsA patients compared with healthy controls, whilst there were no differences between patients with psoriasis and controls. Duration of skin disease was correlated with lower trabecular bone volume and trabecular number, but only in patients with PsA.

To the best of our knowledge, no studies reporting TBS values in patients with psoriasis have been published to date. In our sample, baseline TBS values were lower in men and in patients with mild obesity (defined as a BMI between 30 and 35 Kg/m^2^). There were no differences regarding smoking or dyslipidemia status. After 6 months on adalimumab therapy, only the same differences concerning obese versus nonobese patients were statistically significant. Noteworthily, baseline TBS values were inversely related to some metabolic syndrome features such as blood pressure, obesity parameters (weight, BMI, waist perimeter, and fat percentage measured by DXA), and Castelli index. Blood pressure and obesity measurements were also negatively associated after 6 months of TNF-*α* blockade.

In a study designed to analyze the role of TBS as an indicator of bone quality in diabetes, Kim et al. found that TBS values correlated with metabolic risk factors, such as serum triglycerides, HbA1c, HOMA, fasting plasma glucose, and serum insulin levels [4]. We excluded diabetic patients in our study, and, therefore, the results are not fully comparable. Nevertheless, in our series of nondiabetic patients with moderate-to severe- psoriasis, we observed a significant correlation between insulin resistance (negative) and insulin sensitivity (positive) after 6 months of anti-TNF-*α* therapy.

It is possible that the reduction of the inflammatory burden mediated by the anti-TNF-*α* blockade may, somehow, normalize abnormalities in the glucose metabolism that are directly influenced by inflammation. In keeping with that, we previously observed a significant improvement (*p* = 0.008) of insulin sensitivity after 6 months of adalimumab therapy (QUICKI at baseline: 0.35 ± 0.04 versus 0.37 ± 0.04 at month 6) in our series of nondiabetic patients with moderate-to-severe psoriasis [8].

In line with this assumption, hs-CRP as a marker of inflammation also showed an inverse association with TBS values at baseline and also after 6 months of adalimumab therapy. A similar relationship between TBS and hs-CRP was reported in the aforementioned study by Kim et al. [4].

Adiponectin has been involved in the pathogenesis of the metabolic syndrome and its components, particularly diabetes, obesity, and hypertension [12]. In this regard, in patients with severe rheumatoid arthritis undergoing periodical treatment with anti-TNF-*α*-therapy and ongoing disease activity, low adiponectin levels clustered with metabolic syndrome features that reportedly contribute to atherogenesis in rheumatoid arthritis [13]. In these patients, adiponectin concentrations correlated with triglyceride/HDL cholesterol and total cholesterol/HDL cholesterol ratios, as well as high fasting plasma glucose levels, irrespective of CRP levels and BMI [13]. Moreover, clinical and experimental studies have also found that adiponectin may be an indicator of osteoporotic changes, independent of sex and menopausal status, suggesting that this adipokine may also modulate bone metabolism [14, 15]. Several studies have shown an adverse effect on bone mass, mainly by increasing bone resorption. The pathogenic mechanism has been related to both, a stimulation of the expression of receptor activator of nuclear factor kappa-B ligand (RANKL) and an inhibition of the synthesis of osteoprotegerin by osteoblasts [16]. Moreover, a Th17 cell response has been pointed out in experimental models of collagen-induced arthritis [17]. In a recent meta-analysis, high levels of adiponectin have been negatively correlated with bone mineral density and with a high risk of vertebral fractures, but only in men. High serum leptin levels showed a positive relationship with bone mineral density (BMD) and were associated with a lower risk of fractures. No significant association between resistin, visfatin, or ghrelin and BMD was found [18].

A recent study highlighted a potential regulatory role of adiponectin in cutaneous inflammation in patients with psoriasis [19], and serum levels of this adipokine have been found to increase with psoriasis severity [20]. However, several studies on adiponectin in patients with psoriasis have yielded contradictory results. Because of that, Zhu et al. performed a meta-analysis to clarify this issue and found that adiponectin levels were not significantly different in psoriasis patients compared with controls [21]. Moreover, the effect of TNF-*α* blockade on plasma adiponectin concentrations has been scarcely studied, and results have been discordant [22, 23]. In our patients with moderate-to-severe psoriasis, we observed a significant reduction of adiponectin following adalimumab therapy, and this effect paralleled with a significant improvement in disease severity.

We have also found that, in nondiabetic patients with moderate-to-severe psoriasis, the best set of predictors of changes in bone microarchitecture (measured by the TBS), after a 6-month period on adalimumab, were age, BMI, and serum levels of adiponectin. Thus, we are prompted to hypothesize that this anti-TNF-*α* agent may be beneficial not only in terms of disease severity, but also in terms of bone quality in patients with moderate-to-severe psoriasis by reducing serum adiponectin levels. Besides, this effect is already observed at month 6 of treatment.

## 5. Conclusions

We have shown for the first time that, in nondiabetic patients with moderate-to-severe psoriasis without arthritis, bone microarchitecture, measured by TBS, correlates with some metabolic syndrome features and inflammation, irrespective of sex, age, and duration of psoriasis. These associations were maintained after 6 months of treatment with the anti-TNF-*α* agent, adalimumab. Besides, correlations between TBS and indexes of insulin resistance (HOMA) and insulin sensitivity (QUICKI) were also found after TNF-*α* blockade. Moreover, serum adiponectin levels seem to be an independent variable related to TBS values, after the 6-month period of treatment with adalimumab, but not at baseline. Further studies are needed to improve understanding of the bone microarchitecture and its relationship with metabolic syndrome features, inflammatory markers, and serum adipokines in patients with psoriasis with or without arthritis and in other systemic inflammatory diseases.

## Figures and Tables

**Table 1:** Main baseline epidemiological characteristics of the patients.

Variable	
Sex (M/F), *n* (%)	15 (51.7)/14 (48.3)
Age at study onset (yrs.), m (SD)	38.6 (10.7)
Psoriasis duration (yrs.), median (IQR)	18 (9–27)
Current smokers, *n* (%)	10 (34.5)
Weight (Kg), m (SD)	78.8 (15.5)
BMI (Kg/m^2^), m (SD)	27.5 (3.7)
Waist perimeter (cm), m (SD)	96.1 (10.8)
Systolic BP (mm Hg), m (SD)	120.3 (12.4)
Diastolic BP (mm Hg), m (SD)	75.0 (6.8)
BSA (%), m (SD)	37.9 (16.3)
PASI, m (SD)	18.9 (7.8)
ESR (mm/1st h), median (IQR)	10 (4–21)
hs-CRP (mg/dL), median (IQR)	0.29 (0.07–0.58)
Fat percentage, (%)	32.7 (8.8)
TBS (unitless), m (SD)	1.448 (0.102)

BMI: body mass index. BP: blood pressure. BSA: percent of body surface area affected. PASI: Psoriasis Area and Severity Index. ESR: erythrocyte sedimentation rate. hs-CRP: high-sensitive C-reactive protein. TBS: trabecular bone score.

**Table 2:** Main baseline epidemiological characteristics of the patients.

Variable	Category	TBS		*p*	*p*′
		Baseline	6 months		
		m (SD)	m (SD)		
Sex	Women	1.484 (0.095)	1.487 (0.103)	0.53	0.04
	Men	1.409 (0.097)	1.411 (0.123)	0.78	0.08

Smoking	Yes	1.415 (0.113)	1.414 (0.133)	0.96	0.21
	No	1.466 (0.093)	1.470 (0.108)	0.84	0.23

Dyslipidemia	Yes	1.427 (0.099)	1.426 (0.122)	0.89	0.09
	No	1.495 (0.096)	1.502 (0.092)	0.95	0.08

Obesity	Yes	1.354 (0.078)	1.335 (0.126)	0.61	0.003
	No	1.478 (0.089)	1.487 (0.090)	0.64	0.002

*p* refers to the comparisons between rows and *p*′ between columns for each variable and regarding both study periods (baseline versus 6 months).

**Table 3:** Partial correlations of TBS prior to adalimumab (baseline) and after 6 months of TNF-*α* blockade, in 29 patients with moderate to-severe psoriasis^*∗*^.

Variable	Trabecular bone score (TBS)			
	Baseline		6 months	
	*r*	*p*	*r*	*p*
HOMA	−0.203	0.32	**−0.445**	**0.02**
QUICKI	0.240	0.24	**0.497**	**0.01**
Systolic BP	**−0.424**	**0.03**	**−0.407**	**0.04**
Diastolic BP	**−0.455**	**0.02**	**−0.455**	**0.03**
Total cholesterol	−0.097	0.64	−0.022	0.92
HDL cholesterol	0.345	0.08	0.276	0.17
LDL cholesterol	−0.220	0.28	−0.120	0.56
Triglycerides	−0.176	0.39	−0.130	0.53
Non-HDL cholesterol	−0.246	0.23	−0.141	0.49
Apo-A1	0.189	0.37	0.203	0.32
Apo-B	−0.190	0.36	−0.145	0.48
Lpa	0.100	0.63	0.216	0.30
Castelli index	**−0.411**	**0.04**	−0.329	0.10
Apo-B/Apo-A1	−0.295	0.15	−0.060	0.77
Homocysteine	0.179	0.43	0.055	0.78
ESR	0.093	0.65	−0.024	0.91
HbA1c	−0.330	0.09	0.025	0.91
Insulin	−0.214	0.29	**−0.477**	**0.01**
Glucose	−0.227	0.27	−0.180	0.38
Creatinine	0.292	0.15	0.004	0.98
hs-CRP	**−0.424**	**0.03**	**−0.483**	**0.01**
Weight	**−0.532**	**0.005**	**−0.687**	**0.0001**
BMI	**−0.599**	**0.001**	**−0.764**	**0.0001**
Waist perimeter	**−0.578**	**0.002**	**−0.682**	**0.0001**
BSA	0.146	0.47	−0.147	0.48
PASI	−0.010	0.96	−0.305	0.13
NAPSI hands	0.023	0.91	−0.184	0.39
PGA psoriasis	−0.130	0.53	−0.285	0.17
PASE total score	−0.105	0.61	−0.028	0.89
PASE functional	−0.125	0.54	−0.079	0.70
PASE symptoms	−0.080	0.70	0.017	0.94
Adiponectin	−0.077	0.71	**−0.489**	**0.01**
Leptin	−0.320	0.11	**−0.479**	**0.01**
Resistin	−0.305	0.13	−0.199	0.33
Visfatin	−0.126	0.54	−0.035	0.86
Fat mass (%)	**−0.444**	**0.02**	**−0.560**	**0.003**

^*∗*^Adjusted for sex, age at the study onset, and disease duration.

Significant results are highlighted in bold.
